# Supplementary material for: Quantitative mapping of human hair greying and reversal in relation to life stress
Source: eLife. 2021 Jun 22;10:e67437. doi: 10.7554/eLife.67437 (PMC8219384; doi:10.7554/eLife.67437)
Supplement: Transparent reporting form [file elife-67437-transrepform.docx]

***eLife’s* transparent reporting form**

We encourage authors to provide detailed information *within their submission* to facilitate the interpretation and replication of experiments. Authors can upload supporting documentation to indicate the use of appropriate reporting guidelines for health-related research (see [EQUATOR Network](http://www.equator-network.org/%20)), life science research (see the [BioSharing Information Resource](https://biosharing.org/)), or the [ARRIVE guidelines](http://www.plosbiology.org/article/info:doi/10.1371/journal.pbio.1000412) for reporting work involving animal research. Where applicable, authors should refer to any relevant reporting standards documents in this form.

If you have any questions, please consult our Journal Policies and/or contact us: [editorial@elifesciences.org](mailto:editorial@elifesciences.org).

**Sample-size estimation**

- You should state whether an appropriate sample size was computed when the study was being designed
- You should state the statistical method of sample size computation and any required assumptions
- If no explicit power analysis was used, you should describe how you decided what sample (replicate) size (number) to use

Please outline where this information can be found within the submission (e.g., sections or figure legends), or explain why this information doesn’t apply to your submission:

The rarity of two-colored hairs and absence of prior data on the main outcomes precluded power analysis. This is explained in the “Results” section (page 28) of the manuscript. Hairs were collected from 14 individuals of different ages, ethnicities, and gender, recruited from the United States, Canada, and France. Each hair represents a unique biological event of greying or reversal. A total of 397 hairs were analyzed, including 57 unique greying or reversal events. The relative rarity of two-colored hairs limited the sample size for HPP analysis. Nevertheless, the effect sizes in pigmentation (dark vs white) is unusually large and the densitometric and temporal resolution of the digitization approach provides high precision to map these transitions with high accuracy. Thus, the sample size is sufficient to establish the existence of greying transition and reversal of greying, across a diverse set of individuals and three body regions, as well as their association with behavioral and psychological factors.

For proteomic experiments, which are experimentally challenging and represent a technical tour de force, the sample size is limited but here also the effect sizes are in some cases substantial. To circumvent limitations related to sample size, we opted to perform two independent experiments on different proteomics platforms in independent laboratories, and use converging evidence as a test of robustness (pages 23, 26).

**Replicates**

- You should report how often each experiment was performed
- You should include a definition of biological versus technical replication
- The data obtained should be provided and sufficient information should be provided to indicate the number of independent biological and/or technical replicates
- If you encountered any outliers, you should describe how these were handled
- Criteria for exclusion/inclusion of data should be clearly stated
- High-throughput sequence data should be uploaded before submission, with a private link for reviewers provided (these are available from both GEO and ArrayExpress)

Please outline where this information can be found within the submission (e.g., sections or figure legends), or explain why this information doesn’t apply to your submission:

The information on the number of replicates used for analyses are detailed in the Figure legends (Figures 1, 3-4 and Figure 4-figure supplement 1), “Results” section (pages 23), and in the “Methods” section (pages 10, 16).

Two proteomic experiments were conducted in different laboratories. The degree to which the results were reproducible is detailed in Figure 4, and results for all proteins are available in Supplementary Files 1-3.

With the exception of quality control and detection limits for proteomics experiments detailed in the methods, no data was excluded. Proteomic detection exclusion criteria are outlined in the corresponding Figure legends, in the “Results” section (pages 23, 26 34) and in the “Methods” section (pages 10, 13).

**Statistical reporting**

- Statistical analysis methods should be described and justified
- Raw data should be presented in figures whenever informative to do so (typically when N per group is less than 10)
- For each experiment, you should identify the statistical tests used, exact values of N, definitions of center, methods of multiple test correction, and dispersion and precision measures (e.g., mean, median, SD, SEM, confidence intervals; and, for the major substantive results, a measure of effect size (e.g., Pearson's r, Cohen's d)
- Report exact p-values wherever possible alongside the summary statistics and 95% confidence intervals. These should be reported for all key questions and not only when the p-value is less than 0.05.

Please outline where this information can be found within the submission (e.g., sections or figure legends), or explain why this information doesn’t apply to your submission:

Each Figure legend contains the statistical information and details of tests concerning the data represented in the Figure. P-values and effect sizes can also be found in the “Results” section (pages 24,26-27,29,31,33). A summary of all statistical analysis performed, along with other statistical information can be found in the “Methods” section (page 13-14, 19-21).

(For large datasets, or papers with a very large number of statistical tests, you may upload a single table file with tests, Ns, etc., with reference to sections in the manuscript.)

**Group allocation**

- Indicate how samples were allocated into experimental groups (in the case of clinical studies, please specify allocation to treatment method); if randomization was used, please also state if restricted randomization was applied
- Indicate if masking was used during group allocation, data collection and/or data analysis

Please outline where this information can be found within the submission (e.g., sections or figure legends), or explain why this information doesn’t apply to your submission:

This is a non-interventional observational study. Randomization is not applicable.

**Additional data files (“source data”)**

- We encourage you to upload relevant additional data files, such as numerical data that are represented as a graph in a figure, or as a summary table
- Where provided, these should be in the most useful format, and they can be uploaded as “Source data” files linked to a main figure or table
- Include model definition files including the full list of parameters used
- Include code used for data analysis (e.g., R, MatLab)
- Avoid stating that data files are “available upon request”

Please indicate the figures or tables for which source data files have been provided:

Custom code was developed to create the hair simulation model using R. An interactive implementation was created using Shiny from R Studio, and can be accessed at https://timrain.shinyapps.io/hair/. The source code is downloadable from the App and on GitHub at https://github.com/junting-ren/hair_simulation. All parameters used in the model are listed and explained in Supplementary File 4. Links to access the integrative implementation and source code are also listed in the “Methods” section (pages 17,19).

Functional enrichment analysis of proteomics data was performed using ShinyGo v0.61 (http://bioinformatics.sdstate.edu/go/), an open source platform for which the source code is on GitHub. Protein protein interaction networks were generated, analyzed, and visualized using STRING v.11.0 (https://string-db.org/cgi/input.pl), also freely accessible online. Links for both are listed in the “Methods” section (page 13).

Univariate and multivariate analyses of proteomic signatures, protein abundance levels were processed in R using the Metaboanalyst 3.0 platform (https://www.metaboanalyst.ca), freely available online.

Results for all proteins detected in both proteomic experiments are available in Supplementary Files 1-3.
